# Comparison of Low‐Gluten Diets Rich in Oats or Rice—A 6‐Week Randomized Clinical Trial With Metabolically Challenged Volunteers

**DOI:** 10.1002/mnfr.70076

**Published:** 2025-05-07

**Authors:** Enni Mannila, Petrus Nuotio, Anni Kuosmanen, Suchetana De Storvik, Anna Kårlund, Aija Jukkara, Milla‐Maria Tauriainen, Johanna Närväinen, Marjukka Kolehmainen, Kaisa M. Linderborg

**Affiliations:** ^1^ Food Sciences Department of Life Technologies University of Turku Turku Finland; ^2^ School of Medicine, Institute of Public Health and Clinical Nutrition, Faculty of Health Sciences University of Eastern Finland Kuopio Finland; ^3^ Department of Medicine, Endoscopy Unit Kuopio University Hospital Kuopio Finland; ^4^ VTT Technical Research Centre of Finland Kuopio Finland

**Keywords:** cardiometabolic health, cereals, fiber, gastrointestinal symptoms, metabolic syndrome

## Abstract

Low‐gluten diets (LGD) are also widely followed by people not suffering from coeliac disease. This study compared oats and rice as the main cereal source of an LGD in metabolically challenged volunteers. Volunteers (*n* = 69) were randomly assigned to an LGD, which was either rich in oats or rice, for 6 weeks. Before and after the intervention, concentrations of total cholesterol, LDL‐C, and HDL‐C, triacylglycerols, free fatty acids, glucose, and insulin were measured from fasting plasma samples; the volunteers also completed 4‐day food and stool records, as well as questionnaires related to perceived gastrointestinal discomfort (Gastrointestinal Symptom Rating Scale) and health (RAND‐36). The intervention with oats resulted in a more substantial decrease in LDL‐C (*p*_group × time_ = 0.047), more frequently normal type stool (*p*_group × time_ = 0.010), and bowel movements (*p*_group × time_ = 0.038) than rice (group × time interaction). The rice group experienced more constipation symptoms (*p*_group × time_ < 0.001) than the oat group, possibly due to a lower fiber intake (*p*_group × time_ < 0.001). A greater waist circumference decrease was observed with rice than with oats (*p*_group × time_ = 0.022). Our results suggest that oats improve both biochemical markers of cardiometabolic health and perceived gastrointestinal well‐being compared to rice, thus being a crucial part of a nutritiously adequate LGD.

## Graphical abstract

The aim of this study is to compare oats and rice as the main cereal source of a low‐gluten diet followed by metabolically challenged volunteers. The participants consumed four portions of either oat or rice products daily for 6 weeks and avoided glutinous cereals. The study included measurements of anthropometrics, biochemistry, dietary intake, stool quality, and questionnaires of perceived gastrointestinal symptoms and health. The results suggest oats to be an important part of a low‐gluten diet with a more substantial decrease of LDL‐cholesterol, a higher fiber intake, and improved gastrointestinal symptoms compared to rice.

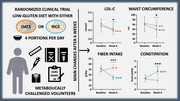

**Abbreviations**
CD: coeliac disease
GF: gluten‐free
GFD: gluten‐free diet
GSRS: Gastrointestinal Symptom Rating Scale
LGD: low‐gluten diet
RAND‐36: RAND 36‐Item Health Survey
SFA: saturated fatty acid
TC: total cholesterol

## 1 Introduction

The gluten‐free diet (GFD) is currently the only treatment for coeliac disease (CD) patients, but nowadays consumers are following GFD also for other reasons. The followers of a GFD avoid all glutinous cereals, such as wheat, barley, rye, contaminated oats, triticale, malt, spelt, kamut, or their hybridized strains [1]. The other than CD‐related reasons for following a GFD include a gluten allergy or intolerance, digestive health reasons, or a GFD being perceived as a “healthier option”, the intention to lose weight, or because another family member follows a GFD [2, 3, 4]. GFD is a demanding diet that requires knowledge from its followers. Silvester et al. [5] found that even CD patients who thought they were strictly following a GFD were actually still consuming gluten in their diet. Many non‐CD patients have started their diet without any patient advocacy group or education from a reliable source and might actually follow—accidentally or intentionally—a low‐gluten diet (LGD) instead of GFD.

Although the GFD may be perceived as healthy, it might actually cause a risk of nutritional deficiencies if not composed carefully [1, 6]. The increase in the number of GFD and LGD followers has vastly increased the currently rising market and development of gluten‐free (GF) products. Nevertheless, GF products are often of poorer nutritional quality compared to their glutinous counterparts, in components such as low content of mineral and fiber content, high proportions of saturated fat, and they typically contain white rice [1]. The lack of fiber in many GFDs is especially problematic, as fiber crucially affects the overall health and the gut microbiota composition and activity [7, 8]. Even with a regular diet, the daily recommendation of fiber intake (25–35 g/day) is often not reached. According to the Global Burden of Disease Study 2017, which included 195 countries, fiber intake was below 15 g/day globally and below 24 g/day in every separate region studied [9]. Similarly, the National FinDiet 2017 survey on the Finnish population reported that approximately 70% did not meet the recommended fiber intake [10]. Our previous studies indicate that by including oats, the lower levels of recommended fiber intake (25 g/day) are reached in GFD [11].

Oats contain the gel‐forming fiber, β‐glucan, which possesses approved health claims in the European Union [12, 13]. In addition to these known effects on lowering blood cholesterol levels, improving postprandial glycaemia, and increasing fecal bulk, oats contain essential minerals and unsaturated fats [6], potentially health beneficial phytochemicals [14], and can modulate the gut microbiota and gut‐brain axis [15]. Oats are also a sustainable crop that can be farmed in different parts of the world and used in various food applications [16, 17]. Oats are typically consumed as whole grain and are available both as gluten free (pure oats kept strictly aside from gluten‐containing grains throughout the production chain) or as generally produced with possible traces of other grains, making oats suitable constituents in GFD and LGD, respectively.

Rice is a gluten‐free staple food for billions of people [18, 19]. Whole grain, that is, brown rice, is beneficial for health, as are other whole grains [20]. The problem is that most of the rice is consumed as white rice, which lacks the nutritional advantages of brown rice [21, 22]. Additionally, the sustainability of rice is compromised due to the fact that rice cultivation uses substantial amounts of water and contributes to greenhouse gas emissions to a great degree [23]. Additionally, rice in Western countries is often consumed at a considerable distance from the production sites [24].

Metabolic syndrome is a combination of interrelated risk factors for type 2 diabetes and cardiovascular disease [25]. The risk factors include obesity (central adiposity), elevated blood pressure, blood triacylglycerol levels, dysglycemia, and low levels of HDL‐C [25]. The prevalence of metabolic syndrome is increasing, along with an increased prevalence of overweight and obesity globally [26]. Metabolic syndrome can be improved with lifestyle changes such as a healthier diet [27]. In addition to metabolic syndrome, cardiovascular diseases, inflammatory bowel diseases, and other gastrointestinal issues are common health related problems in countries following a western lifestyle [26, 28].

Nutrient intake of CD patients consuming a GFD may shift towards more saturated fats and lower fiber intake, which can be an indirect cardiometabolic risk factor [29]. Indeed, CD patients have been shown to have a higher risk of developing metabolic disease and hepatic steatosis after following a GFD [30]. However, contrary to the above‐mentioned studies, previous reviews conclude either positive [31] or unclear effects [32] regarding the cardiometabolic risk of a GFD on CD patients. Regarding the evidence on the health benefits of a GFD or LGD for those without gluten‐related disorders, the few previous studies are inconsistent [33, 34, 35, 36, 37]. However, there is some evidence of a better lipid profile, a healthier waist circumference, and improved bloating symptoms for followers of GFDs and LGDs [37, 38, 39].

As previously reviewed, Schmucker et al. [36] showed that there is a slightly increased risk to develop type 2 diabetes with a lower gluten intake (0–4.3 g/day) compared to a higher gluten intake (5.9–38.4 g/day). On the contrary, Dhruva et al. [35] found slight improvements in overall cardiac risk factors, insulin resistance, and weight after following a GFD. However, the gluten intake has not always been associated with cardiovascular disease or metabolic syndrome [33, 37]. It has been concluded that gluten intake is not a major contributor to the metabolic health of non‐CD people, and rather other factors affect the risks for metabolic disease [33]. However, by including oats, it would be possible to improve cardiometabolic health regardless of the dietary background of people with a risk of metabolic syndrome [40].

Because of the wide use of GFDs and LGDs and the previous inconsistent results, it is important to understand how oats and rice, both naturally gluten‐free staple cereals, influence the nutritional quality of these diets and their health impact, especially on people without CD and who are at risk of developing a metabolic syndrome. In previous studies with participants at risk of metabolic syndrome, the focus has been either on the effects of a GFD [41, 42], oat β‐glucan [43, 44, 45], or oat phytochemicals [46]. In our study, we compared the LGDs rich in either oats or rice on the intake of nutrients and perceived health and cardiometabolic risk factors. Our hypothesis was that oats would improve the nutritional quality of the LGD compared to rice, as well as enhance the perceived health and cardiometabolic status. To our knowledge, this is the first study to compare two naturally gluten‐free cereals with their cardiometabolic effects as part of a popular LGD on people without gluten‐related disorders.

## 2 Experimental Section

The dietary intervention research described here belongs to the larger study OAT‐GUT‐BRAIN, which aims to reveal the comprehensive health effects of oats in the context of an LGD followed by metabolically challenged volunteers. The intervention was conducted at the Institute of Public Health and Clinical Nutrition, School of Medicine, Faculty of Health Sciences at the University of Eastern Finland in Kuopio. The study received a positive statement from the Research Ethics Committee of the Northern Savo Hospital District (1116/2022 OAT‐GUT‐BRAIN) and was registered in ClinicalTrials.gov (Identifier: NCT05526092). The participants provided a written informed consent prior to participation, and they were able to withdraw from the study at any point.

### 2.1 Study Design

**FIGURE 1:**
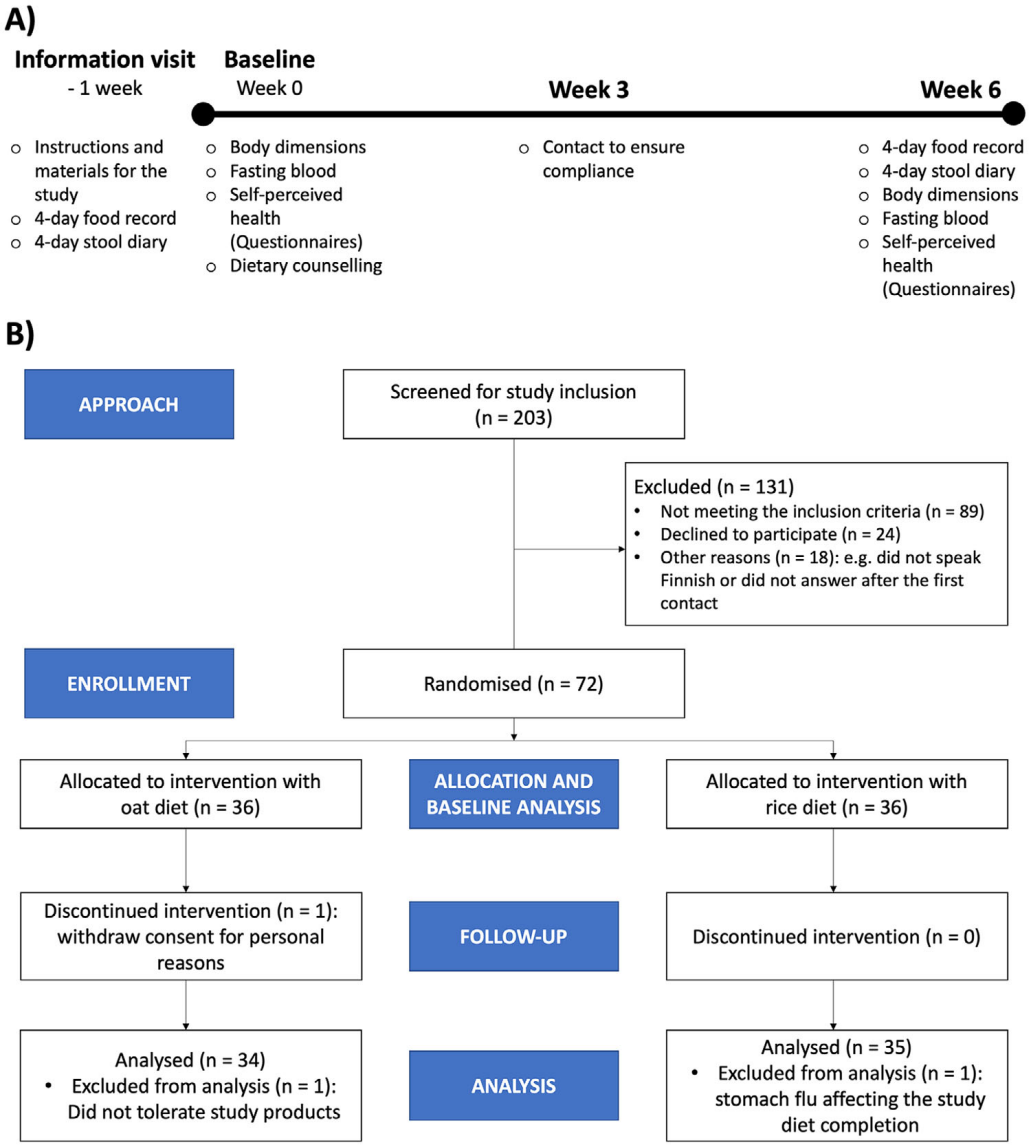
(A) Study design (B) The CONSORT flow diagram.

The study design and CONSORT flow are presented in Figure 1. The study was a randomized single‐blinded clinical trial in which metabolically challenged individuals followed a low‐gluten oat or rice‐rich diet for 6 weeks (range 5‒9 weeks). The participants were randomly allocated into oat and rice diet groups (Williams Design in Compusense22 software, Compusense, Guelph, Canada). To elucidate the impact of a genuine situation among common consumers, the study was single‐blinded so that the volunteers knew they were to eat either oats or rice and avoid the other. One week prior to the first actual study visit, the participants were given instructions on the study records and sample collection. On the first study day, the participants returned completed food and stool records, gave a fasting blood sample, filled in the questionnaires, received dietary counseling for the study diet, and were provided with the study food items. In the third week, participants were contacted to ensure the suitability of the diet and their compliance with the diet, which was also confirmed from their product diaries and food frequency questionnaires. The participants had the possibility to contact the researcher via email and phone every weekday. In the last study visit, after the 6‐week intervention, the participants went through similar blood sampling and questionnaires as in the first study visit.

### 2.2 Study Subjects

The inclusion criteria for the eligibility of participants were the following: being overweight or obese (BMI 24–38), age of 30–68 years, and having either a total cholesterol of plasma over 5 and a high LDL‐C (> 3 mmol/L) and/or low HDL‐C (men < 1.0 mmol/L; women < 1.2 mmol/L), or high blood pressure (≥ 140/90 mmHg), or both. The exclusion criteria included pregnancy and lactation, heavy smoking, a recent course of antibiotics, and cholesterol medication. Balanced medication for hypertension or hypo‐/hyperthyroidism, hormone replacement therapy, and inhaled medicines were allowed, while participants on other types of regular medication were excluded. In total, 203 participants were screened for inclusion in the Northern Savo region of Finland from September 2022 to May 2023 (Figure 1B). Of these, 131 were excluded for not meeting the inclusion criteria or declining to participate. Seventy‐two participants were randomly divided into oat (36 persons) and rice (36 persons) groups. One person from the oat group withdrew consent to participate, and the remaining 71 participants completed the study. The participants contacted the researcher for any adverse effects, and the medical doctor responsible was consulted. One participant was excluded due to not tolerating the study products, and another due to acute gastroenteritis affecting the study diet completion. The analyses were made for 69 participants in total, except for individual missing data points mentioned in Sections 2.4, 2.5.1, and 2.5.3. The baseline characteristics of the study participants are presented in Table 1.

**TABLE 1:** Baseline characteristics of the study participants as mean (standard deviation).

	All participants (*n* = 69)
Female/Male/Other	46/22/1
Age (years)	51.2 (10.5)
Weight (kg)	83.0 (12.7)
BMI (kg/m^2^)	29.1 (3.1)
Waist circumference (cm)	96.2 (8.6)
Blood pressure (mmHg)	
Systolic	130.3 (16.0)
Diastolic	86.2 (9.5)
Total cholesterol (mmol/L)	5.58 (1.09)
LDL cholesterol (mmol/L)	3.80 (0.93)
HDL cholesterol (mmol/L)	1.52 (0.43)
Triacylglycerols (mmol/L)	1.27 (0.51)
Free fatty acids (mmol/L)	0.41 (0.12)
Glucose (mmol/L)	5.71 (0.50)
Insulin (mU/L)	8.92 (4.94)

### 2.3 Study Diet and Products

Both the low‐gluten oat‐ and rice‐rich diets were designed to contain approximately 100 g of oats or rice per day and to cover 500 kcal of daily energy intake from either oats or rice. The oat group was designed to consume 6 g of oat β‐glucan per day. Since the cereal products are the main source of gluten, the participants were asked to change their habitual cereal products to oat or rice products and to consume at least four portions of oats or rice according to their group allocation. The oat group consumed oats as the main cereal and avoided rice, and the rice group consumed rice, avoiding oats. A versatile selection of commercial oat and rice products (listed in Tables S1 and S2) was provided for the participants. These included, for example, porridge flakes, cereals, oat grains and rice, snack bars, and rice cakes. The participants were also allowed to use other commercial or self‐prepared products according to their diet group, and they were advised to report the additional portions. They were requested to maintain their other dietary habits and physical activity unchanged during the intervention. For better 6‐week compliance, participants were allowed to eat freely either for three meals or one whole day per week. The participants received oral and written instructions for the study diet from a trained researcher on the first study day. The compliance was reviewed in the mid‐study checkup.

### 2.4 Blood Sampling and Anthropometric Measurements

Blood samples were collected, and the weight, height, blood pressure, and waist circumference were measured after an overnight fast on the first and the last study visit (Figure 1A). The participants were instructed to abstain from vigorous exercise and alcohol consumption for 48 h prior to the visit. The participants were asked to complete a food record about the foods and drinks during the evening before the start of fasting and eat similarly on the evening before both the fasting blood samples. Blood pressure was measured twice, with a 1‐min break between measurements. One participant started hypertension medication during the intervention, and this subject's results were excluded from blood pressure data analysis. Venous blood samples were collected in 9 mL EDTA VACUETTE tubes that were mixed 6–10 times and kept on ice prior to centrifugation at 2400 × *g* at +4°C for 10 min. The plasma samples were stored at −85°C within 30 min after the sampling. Fasting plasma total cholesterol (TC), low‐density (LDL‐C) and high‐density lipoprotein cholesterol (HDL‐C), serum triacylglycerols, free fatty acids, serum glucose, and insulin concentration were analyzed as previously described [47].

### 2.5 Records and Questionnaires

The participants were given oral and written instructions on how to complete records and questionnaires. The records and the questionnaires needed to be completed before the first and the last study visits.

#### 2.5.1 Food Record

The participants were asked to keep food records during three consecutive weekdays and one weekend day. The nutrient calculations for the 4‐day food records were performed using the AivoDiet software (AivoDiet 2.2.0.0, Mashie, Malmö, Sweden), based on national and international analyses, and international food composition tables (fineli.fi). Food records were reviewed by a trained researcher together with the participants. The food records of 69 participants were analyzed for nutrient intake. One participant's 6th‐week dietary assessment was conducted through a 2‐day dietary interview, but it was included. One food record on the 6th week was excluded due to incomplete reporting.

#### 2.5.2 Product Diaries

Product diaries were used to follow compliance with the consumption of the target foods provided to the participants. The amount of the consumed target foods was calculated based on these records. The participants marked the consumed products as simple marks of used portions of a specified product and aimed for four portions per day. Participants also recorded the free‐eating days or meals and other consumed gluten containing products eaten during the intervention. The number of eaten study products was calculated as g/day, and similar products were categorized together. β‐glucan intake in the oat group was calculated from the fiber content on the oat product labels, assuming 40% of the fiber to be β‐glucan [48].

#### 2.5.3 Bristol Defecation Record

The changes in bowel movement frequency and stool consistency were recorded using a defecation record with the 7‐step Bristol stool chart for the same 4 days as the food record [49]. The participant chose the dominant stool type for each defecation and recorded the time of defecation. The Bristol types were then categorized as hard (types 1 and 2), normal (types 3, 4, and 5), or loose stools (types 6 and 7), and the percentage of occurrence for both intervention groups was determined [50]. One defecation record of the 6th week was not obtained from one participant in the oat group.

#### 2.5.4 Questionnaires on Gut Symptoms and General Health

The participants evaluated their subjective experience regarding their gastrointestinal symptoms with the Gastrointestinal Symptoms Rating Scale (GSRS) [51, 52, 53] and their general health with the RAND‐36 Survey, derived from the Short Form‐36 Health Survey (SF‐36) [54, 55, 56]. The GSRS results were rated according to the severity of a symptom and divided into stomachache, bloating, flatulence, constipation, and diarrhea [52, 57]. The RAND‐36 answers were rated according to the instructions for indices that were then divided into scales of physical and social functionality, role limitations caused by physical and emotional health problems, bodily pain, general health perception, vitality, and mental health [56].

### 2.6 Statistical Analyses

The required number of study participants was estimated based on serum total cholesterol as an endpoint. Based on the power calculation on the effects of whole grain‐rich diets on plasma lipid profile [58], we aimed to randomize 53 subjects into each group. This was expected to provide 80% power to detect a minimal difference of 0.5 mmol/L in serum total cholesterol between groups, assuming a within‐patient standard deviation of 0.9 mmol/L (two‐sided *α* = 0.05, http://hedwig.mgh.harvard.edu/sample_size/). Per protocol analysis including all 69 completers was employed. The primary analyses (changes in dietary intake, clinical variables, GSRS/RAND‐36 questionnaires, and stool record) were performed using linear mixed models (LMM, R packages lme4 v1.1‐30 [59] and lmerTest v3.1‐3 [60]). Models were fitted using a restricted maximum likelihood (REML) method whilst ignoring missing observations. Response variables were Box‐Cox transformed [61] to address their generally skewed distributions prior to statistical analyses. Assumptions were tested by plotting residual and predicted values and by visually inspecting residual Q‐Q plots to test homogeneity of variances and normality of residuals, respectively. Within each diet group, models were run using the outcome of interest as the response variable, subject identifier as a random effect (intercept), and timepoint as a fixed effect. To compare changes between the diet groups (i.e., diet × timepoint interaction), data from both oat and rice groups were pooled together, and similar models were run using timepoint, diet, and diet × timepoint interaction as fixed effects. Confidence intervals (95%) for within‐group changes were calculated from untransformed delta variables (i.e., the difference between baseline and Week 6 raw values) to preserve the original units. Box‐Cox‐transformed variables with absolute skewness > 1 had non‐normal residuals and were thus re‐analyzed and reported using Wilcoxon signed‐rank tests. Paired or unpaired tests were used for within‐group changes and between‐group differences in changes (delta variables), respectively. Both LMMs and Wilcoxon tests detected the same exact phenomena as significant at the *α* = 0.05 level, highlighting the robustness of LMMs for slight assumption violations [62]. Wilcoxon effect sizes (*r*) were reported for all variables to assess the magnitude and importance of both within‐group and between‐group results. Wilcoxon effect sizes were interpreted as commonly in literature: 0.10 to < 0.3: small effect, 0.30 to < 0.5: moderate effect, and ≥ 0.5: large effect. Daily portions were compared between the diets using a Wilcoxon signed‐rank test. All tests were two‐tailed, and *p* values < 0.05 were considered statistically significant. Statistical analyses were performed using R v4.2.2 (R Core Team 2022), and results were visualized using GraphPad Prism (Version 10.1.2, build number 324).

## 3 Results

### 3.1 Compliance

The compliance of the participants was evaluated orally and through the food records and product diaries. Both groups met the objective of a minimum of 4 portions per day. However, the oat group consumed significantly more oat product portions a day, than the rice group rice products (Table 2). In addition to the provided products, oat products such as 100% oat bread were consumed by the oat group participants, while the rice group ate mostly only the provided rice products. The oat group consumed over 7 g of β‐glucan daily (Table 2).

**TABLE 2:** The daily portions of each category in both diet groups (mean ± standard deviation).

	Group		
Portions/day	Oat *n* = 34	Rice *n* = 35	*p*
Provided products	3.2 ± 1.2	4.1 ± 1.0	< 0.001
Other	2.3 ± 1.1	0.3 ± 0.3	< 0.001
Total	5.6 ± 1.6	4.4 ± 1.0	< 0.001
Oat β‐glucan (g/day)			
from provided products	4.6 ± 1.7		
from other	2.5 ± 1.2		
Total	7.0 ± 2.2		

*Note*: The participants aimed for a minimum of 4 portions per day. Oat: provided products = porridge/flakes, cooked oat, snack bar, cookie, granola; other = 100% oat bread and pasta, bran, oatgurt, other used oat products. Rice: provided products = porridge/flakes, cooked rice (as such, in sushi or in Karelian pastries), rice cakes, rice crispies; other = rice noodles, rice bread, gluten free pasta made with rice flour, rice pudding, other used rice products. *p* value from Wilcoxon signed‐rank test. The daily intake of β‐glucan was calculated from the fiber information of the product labels. Example portions and foods are presented in Table S1, and the usage of the Other category in Table S2.

### 3.2 Intake of Energy and Nutrients During the Oat and Rice Diet Interventions

**FIGURE 2:**
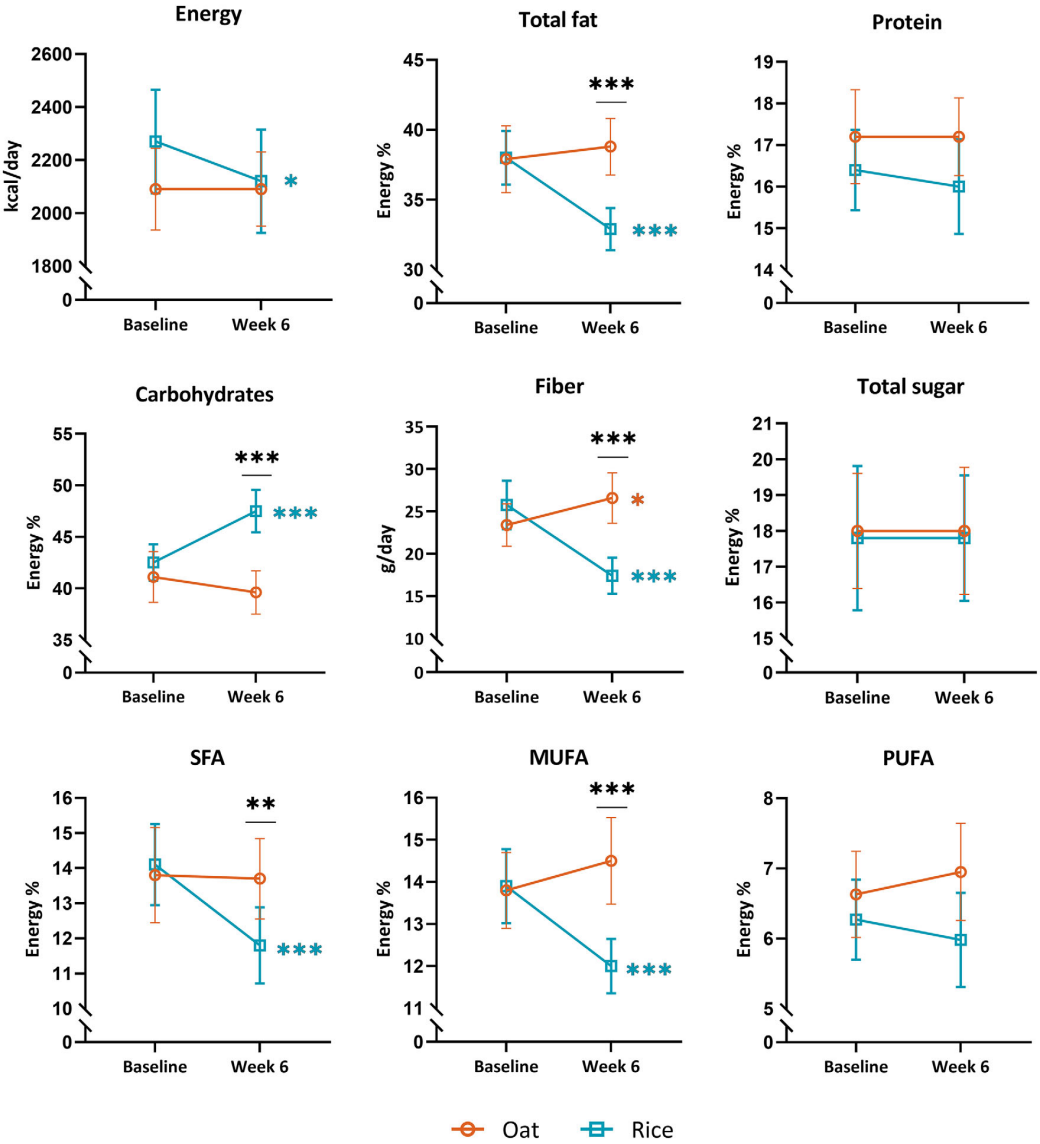
Macronutrients in the oat (orange, circle) and in the rice group (blue, square) as mean with a 95% confidence interval. Energy%, percent of total energy intake. MUFA, monounsaturated fatty acid; PUFA, polyunsaturated fatty acid; SFA, saturated fatty acid. *p < 0.05, **p < 0.01, ***p ≤ 0.001. Complete results are shown in Table S3.

**FIGURE 3:**
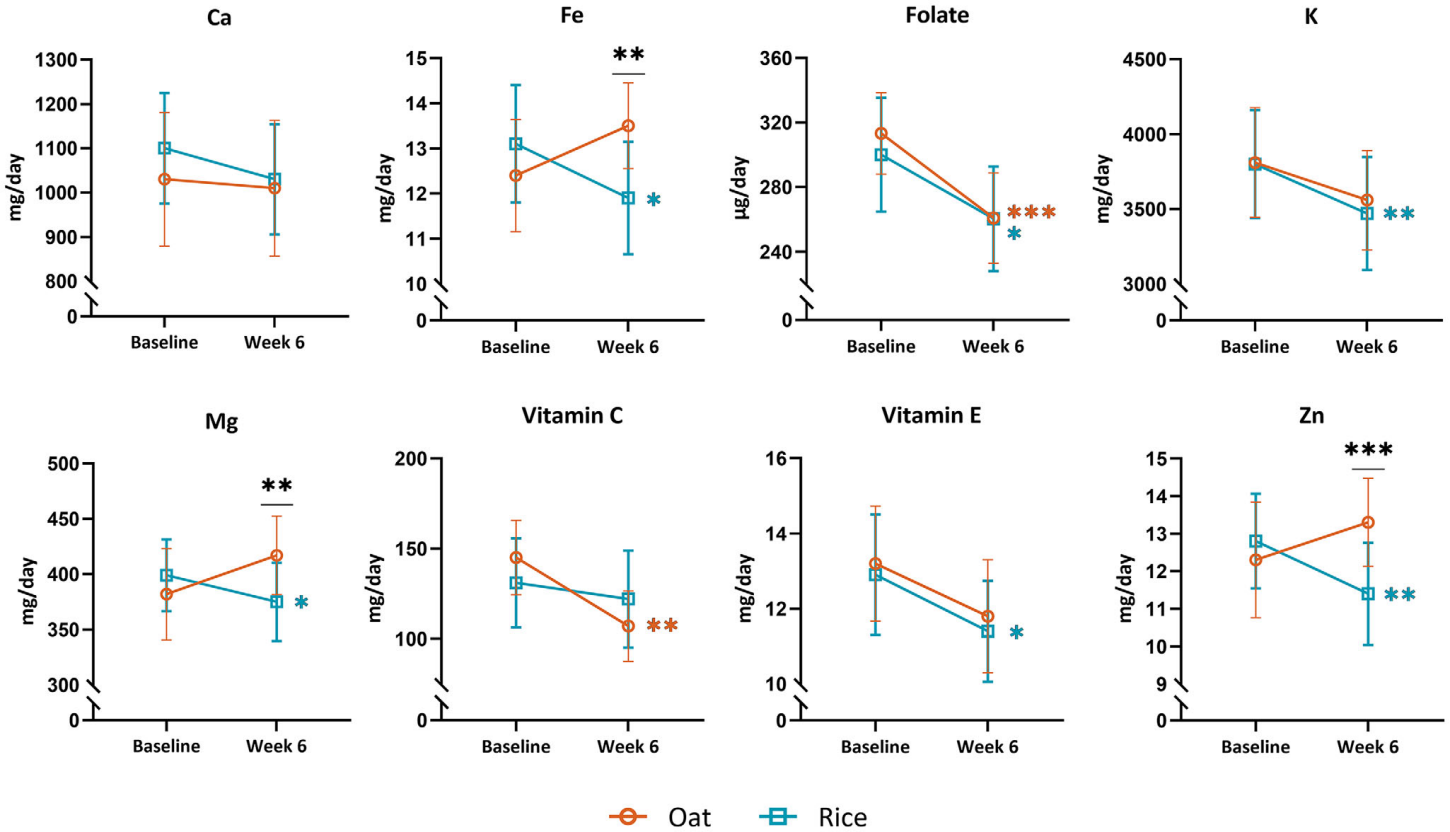
Micronutrients in the oat (orange, circle) and in the rice group (blue, square) as mean with a 95% confidence interval. The nutrients are shown as mg/day, but folate is shown as µg/day. Ca, calcium, Fe, iron; K, potassium; Mg, magnesium; Zn, zinc. *p < 0.05, **p < 0.01, ***p ≤ 0.001. Complete results are shown in Table S3.

During the intervention, the intake of several nutrients, namely energy from carbohydrates and fat, including saturated (SFAs) and monounsaturated fatty acids (MUFAs), as well as fiber (as grams per day and per MJ), differed significantly between the oat and rice groups, with moderate to large effect sizes (0.30 to 0.61; Figure 2, Table S3). Additionally, intakes of magnesium, iron, and zinc differed between the groups during the intervention, with small to moderate (0.29 to 0.38) effect sizes (Figure 3, Table S3).

Energy intake from carbohydrates increased in the rice group (+4.79 ± 6.14 E%, *p*_time_ < 0.001), while it remained unchanged in the oat group (*p*_time_ > 0.05; *p*_group × time_ < 0.001). Fiber intake increased moderately in the oat group (+3.19 ± 8.42 g/day, effect size 0.35, *p*_time_ = 0.048) and decreased substantially in the rice group (−8.35 ± 7.11, effect size 0.80, *p*_time_ < 0.001; effect size between groups 0.61, *p*_group × time_ < 0.001). Energy intake from sugar did not change during the intervention (*p*_group × time_ > 0.05 and *p*_time_ > 0.05 in both the groups). Energy intake from total fat, SFAs, and MUFAs decreased markedly within the rice group (*p*_time_ < 0.001, effect size 0.51 to 0.68), but remained unchanged within the oat group (*p*_time_ > 0.05; *p*_group × time_ < 0.01). The intakes of magnesium, iron, and zinc differed between the groups significantly (*p*_group × time_ = 0.007, 0.003, 0.001, respectively) with a small to moderate effect: within the oat group, the intake tended to increase (*p*_time_ = 0.075, 0.056, 0.074, respectively), and within the rice group, it decreased significantly (*p*_time_ = 0.020, 0.020, 0.004, respectively). Additionally, the intakes of folate and vitamin C decreased substantially within the oat group (−52.46 ± 84.63 µg/day, *p*_time_ < 0.001, and −37.59 ± 66.06 mg/day, *p*_time_ = 0.002, respectively), and there was a tendency for slightly decreased intake of vitamin E (*p*_time_ = 0.070). Within the rice group, the total energy intake (−186 ± 380 kcal/day, *p*_time_ = 0.011), and the intake of vitamin E (−1.65 ± 4.36 mg/day, *p*_time_ = 0.033), folate (−42.81 ± 113.74 µg/day, *p*_time_ = 0.017), and potassium (−387.67 ± 717.41 mg/day, *p*_time_ = 0.003) decreased moderately. Furthermore, there was a tendency for a slightly decreased calcium intake (−91.69 ± 274.70 mg/day, *p*_time_ = 0.069) in the rice group.

### 3.3 Clinical and Biochemical Measurements

The results of the clinical and biochemical measurements are shown in Table 3. Both diets reduced waist circumference with a significantly larger decrease in the rice group (oat: −1.0 ± 1.8 cm; rice: −2.1 ± 2.3 cm, *p*_group × time_ = 0.022). Within both the groups, weight, BMI, and waist circumference decreased significantly (*p*_time_ = 0.002 to 0.005 in the oat group, and *p*_time_ < 0.001 in the rice group), with effect sizes ranging from moderate to large. The LDL‐C reduction was significantly different between the diet groups (effect size 0.27 between the groups, *p*_group × time_ = 0.047), with a large reduction in the oat group (−0.41 ± 0.49 mmol/L, effect size 0.65, *p*_time_ < 0.001) and a small reduction in the rice group (−0.17 ± 0.50 mmol/L, effect size 0.25, *p*_time_ = 0.056). Additionally, TC was significantly reduced within the oat group (−0.35 ± 0.62, effect size 0.52, *p*_time_ = 0.003). No significant differences were found between or within the study groups regarding blood pressure, HDL‐C, triacylglycerols, free fatty acids, glucose, or insulin levels. The oat group showed a tendency toward a decrease in free fatty acid levels (−0.04 ± 0.14 mmol/L, *p*_time_ = 0.063).

**TABLE 3:** Participants’ clinical and biochemical results of the baseline and at the 6th week presented as mean (standard deviation) with a 95% confidence interval for the change.

	Oat (*n* = 34)	Rice (*n* = 35)		
Female/Male/Other	24/10/0	22/12/1		
Age (years)	53 (10)	49 (10)		

	Baseline	Week 6	Δ (SD)	Δ 95% CI	ES_time_	*p*_time_	Baseline	Week 6	Δ (SD)	Δ 95% CI	ES_time_	*p*_time_	ES_group × time_	*p*_group × time_
Weight (kg)	82.0 (12.0)	81.5 (12.2)	−0.5 (1.2)	−0.9 to −0.1	0.44	**0.005**	84.0 (13.4)	83.1 (13.3)	−0.9 (1.4)	−1.4 to −0.4	0.55	**< 0.001**	0.13	0.3
BMI (kg/m^2^)	29.1 (3.1)	28.9 (3.1)	−0.2 (0.4)	−0.3 to −0.0	0.42	**0.004**	29.1 (3.1)	28.8 (3.1)	−0.3 (0.5)	−0.5 to −0.1	0.55	**< 0.001**	0.13	0.2
Waist circumference (cm)	96.5 (8.4)	95.4 (8.4)	−1.0 (1.8)	−1.7 to −0.4	0.52	**0.002**	96.0 (8.9)	93.8 (9.0)	−2.1 (2.3)	−2.9 to −1.4	0.69	**< 0.001**	0.25	**0.022**
Blood pressure (mmHg) ^a^														
Systolic	134.8 (19.1)	131.6 (18.0)	−3.2 (11.2)	−7.1 to 0.8	0.27	0.10	126.1 (11.2)	124.2 (10.9)	−1.9 (8.5)	−4.9 to 1.0	0.20	0.2	0.07	0.7
Diastolic	88.6 (10.5)	87.9 (10.7)	−0.7 (5.5)	−2.6 to 1.2	0.11	0.4	83.9 (7.8)	82.9 (9.3)	−1.0 (6.0)	−3.0 to 1.1	0.16	0.3	0.04	0.8
Cholesterol, total (mmol/L)	5.60 (1.07)	5.25 (0.88)	−0.35 (0.62)	−0.57 to −0.13	0.52	**0.003**	5.55 (1.12)	5.39 (0.95)	−0.17 (0.64)	−0.39 to 0.05	0.08	0.2	0.22	0.2
LDL‐C (mmol/L)	3.87 (0.84)	3.46 (0.63)	−0.41 (0.49)	−0.59 to −0.24	0.65	**< 0.001**	3.73 (1.02)	3.55 (0.92)	−0.17 (0.50)	−0.34 to 0.00	0.25	0.056	0.27	**0.047**
HDL‐C (mmol/L)	1.50 (0.44)	1.49 (0.43)	−0.01 (0.15)	−0.07 to 0.04	0.12	0.5	1.53 (0.43)	1.55 (0.43)	0.02 (0.20)	−0.05 to 0.09	0.11	0.6	0.10	0.4
Triacylglycerols (mmol/L)	1.27 (0.47)	1.31 (0.64)	0.04 (0.41)	−0.10 to 0.18	0.03	> 0.9	1.26 (0.55)	1.22 (0.62)	−0.04 (0.42)	−0.18 to 0.11	0.14	0.3	0.01	0.5
Free fatty acids (mmol/L)	0.41 (0.14)	0.37 (0.11)	−0.04 (0.14)	−0.09 to 0.00	0.24	0.063	0.40 (0.10)	0.39 (0.14)	−0.01 (0.13)	−0.05 to 0.04	0.16	0.6	0.05	0.3
Glucose (mmol/L)	5.72 (0.41)	5.66 (0.38)	−0.06 (0.39)	−0.20 to 0.07	0.30	0.4	5.71 (0.58)	5.69 (0.53)	−0.02 (0.38)	−0.15 to 0.11	0.05	0.8	0.10	0.6
Insulin (mU/L)	9.28 (5.55)	9.30 (5.05)	0.02 (4.62)	−1.59 to 1.64	0.00	0.9	8.58 (4.33)	9.19 (5.54)	0.61 (4.49)	−0.93 to 2.16	0.01	0.5	0.01	0.7

*Note*: The bolded values present significances at the level *p* < 0.05, *p* values obtained from linear mixed model, *p*_time_ standing for within‐group change over time and *p*_group × time_ for between‐group difference in changes (interaction: group × time).

Abbreviations: 95% CI, confidence interval; ES, Wilcoxon effect size (*r*); SD, standard deviation.

^a^ Oat group *n* = 33, excluded one person who started blood pressure medication during the intervention.

### 3.4 Defecation Frequency and Stool Type

At the end of the intervention, the bowel movement frequency was higher in the oat group than in the rice group (1.57 ± 0.65 and 1.24 ± 0.50 bowel movements/day, respectively, *p*_group × time_ = 0.038, Table 4.) Although no significant changes within the groups were seen, there was a tendency within the oat group for an increase in bowel movements between the beginning and the end of the intervention (+0.17 ± 0.62 bowel movements/day, *p*_time_ = 0.083). The normal stool per 4 recorded days differed between the groups (*p*_group × time_ = 0.010, Table 4.) Within the groups, there was a tendency for a higher amount of normal stool within the oat group (+0.85 ± 2.64 normal stool/4 days, *p*_time_ = 0.082) and for a lesser amount within the rice group (−0.91 ± 0.64 normal stool/4 days, *p*_time_ = 0.056) compared to the beginning of the intervention. These changes were small according to the effect sizes (< 0.30). A visualization of the percentage differences of the categorized stool types for the 4 days between and within the groups is presented in Figure S1.

**TABLE 4:** Categorized stool types during the recorded 4 days and bowel movement frequency per day in the oat and rice intervention groups as mean (standard deviation) with a 95% confidence interval for the change.

	Oat						Rice							
	Baseline	Week 6					Baseline	Week 6						
Stool type /4 days	Mean (SD)	Mean (SD)	Δ (SD)	Δ 95% CI	ES_time_	*p*_time_	Mean (SD)	Mean (SD)	Δ (SD)	Δ 95% CI	ES_time_	*p*_time_	ES_group × time_	*p*_group × time_
No stool	0.21 (0.54)	0.09 (0.38)	−0.12 (0.42)	−0.27 to 0.03	0.30	0.17^*^	0.29 (0.52)	0.40 (0.74)	0.11 (0.72)	−0.13 to 0.36	0.11	0.4^*^	0.16	0.063^*^
Hard stool	0.35 (1.01)	0.48 (1.23)	0.12 (1.14)	−0.28 to 0.53	0.12	0.4^*^	0.34 (0.59)	0.97 (2.27)	0.63 (2.24)	−0.14 to 1.40	0.17	0.15^*^	0.04	0.7^*^
Normal stool	4.21 (2.01)	5.03 (2.28)	0.85 (2.64)	−0.09 to 1.78	0.26	0.082	4.26 (2.03)	3.34 (1.81)	−0.91 (2.64)	−1.82 to −0.01	0.29	0.056	0.27	**0.010**
Loose stool	1.06 (1.84)	0.70 (1.07)	−0.36 (1.82)	−1.01 to 0.28	0.12	0.3	0.57 (1.01)	0.49 (0.95)	−0.09 (1.07)	−0.45 to 0.28	0.09	0.7	0.02	0.7
Frequency, BM/day	1.40 (0.57)	1.57 (0.65)	0.17 (0.62)	−0.05 to 0.39	0.21	0.083	1.30 (0.51)	1.24 (0.50)	−0.07 (0.37)	−0.19 to 0.06	0.13	0.2	0.17	**0.038**

*Note*: Oat group baseline *n* = 34, Week 6 *n* = 33; rice group *n* = 35. The bolded values present significances at the level *p* < 0.05, *p* values obtained from linear mixed model, *p*_time_ standing for within‐group change over time and *p*_group × time_ for between‐group difference in changes (interaction: group × time). No stool: reported no defecation; Hard stool: Bristol types 1 and 2; Normal stool: Bristol types 3, 4, 5; Loose stool: Bristol types 6, 7; BM: Bowel movements.

Abbreviations: 95% CI, confidence interval; ES, Wilcoxon effect size (*r*); SD, standard deviation.

* *p* value from the Wilcoxon signed‐rank test due to skewed data.

### 3.5 Perceived Gastrointestinal and General Well‐Being

**FIGURE 4:**
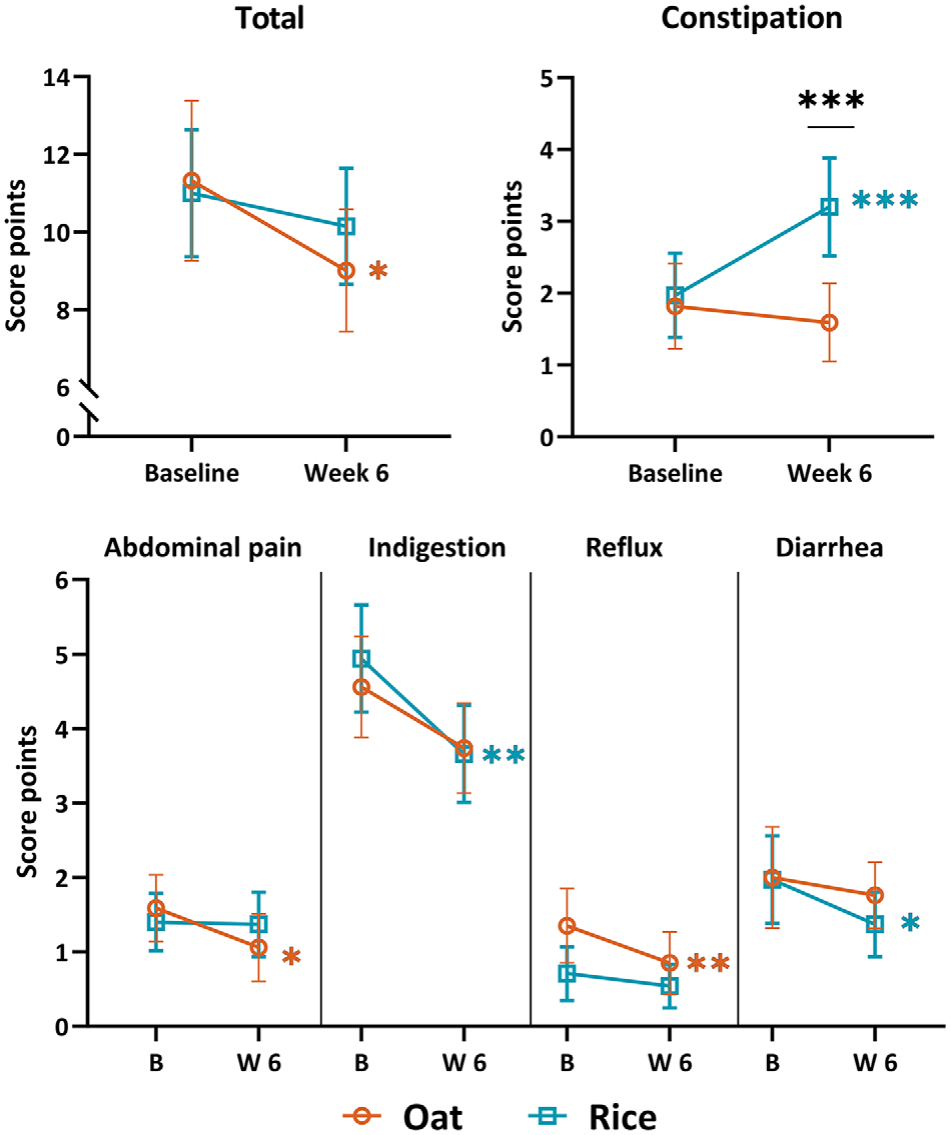
Gastrointestinal Symptoms Rating Scale (GSRS) score results for the oat (orange, circle) and the rice group (blue, square) as mean with a 95% confidence interval. A higher score refers to more symptoms. B, baseline, W 6, Week 6. *p < 0.05, **p < 0.01, ***p ≤ 0.001. Complete results are shown in Table S4.

In GSRS, the constipation score differed moderately between the groups, being higher in the rice group than in the oat group (3.2 ± 2.0 points and 1.6 ± 1.6 points, respectively, effect size between groups 0.40, *p*_group × time_ < 0.001, Figure 4, Table S4). Additionally, the constipation symptoms increased substantially within the rice group (+1.2 ± 2.0 points, effect size 0.53, *p*_time_ = 0.001). Within the oat group, the total symptoms (−2.3 ± 5.9 points, *p*_time_ = 0.033), abdominal pain (−0.5 ± 1.7 points, *p*_time_ = 0.018), and reflux scores (−0.5 ± 1.0 points, *p*_time_ = 0.005) improved, and the indigestion score tended to improve (−0.8 ± 2.4 points, *p*_time_ = 0.050). Within the rice group, significant improvements were in indigestion (−1.3 ± 2.7 points, *p*_time_ = 0.008) and diarrhea scores (−0.6 ± 1.4 points, *p*_time_ = 0.042). Effect sizes for these changes were small to moderate. The results of the GSRS are fully shown in Table S4.

The RAND‐36 did not show any between‐group differences in changes either in general well‐being as the total score of the questionnaire or in the well‐being subcategories (Table 5). However, the total score increased within both the groups (+2.8 ± 5.5 points within the oat group, *p*_time_ = 0.002; and +2.0 ± 5.7 points within the rice group, *p*_time_ = 0.033). The effect size for the total score was considered as large (0.53) with oats and moderate (0.36) with rice. Within the oat group, the perception of emotional well‐being also improved moderately (+4.1 ± 9.7 points, effect size 0.44, *p*_time_ = 0.013).

**TABLE 5:** Results of RAND‐36 questionnaire scores presented as mean (standard deviation) with 95% confidence interval for the change.

	Oat						Rice							
	Baseline	Week 6					Baseline	Week 6						
RAND‐36 Score	Mean (SD)	Mean (SD)	Δ (SD)	Δ 95% CI	ES_time_	*p*_time_	Mean (SD)	Mean (SD)	Δ (SD)	Δ 95% CI	ES_time_	*p*_time_	ES_group × time_	*p*_group × time_
Total	82.5 (10.3)	85.3 (9.35)	2.8 (5.5)	0.87 to 4.69	0.53	**0.002** ^*^	83.1 (8.3)	85.1 (7.3)	2.0 (5.7)	0.04 to 3.99	0.36	**0.033** ^*^	0.10	0.2^*^
Physical functioning	92.7 (9.5)	93.1 (8.35)	0.4 (5.3)	−1.40 to 2.28	0.13	0.8^*^	94.3 (7.6)	95.3 (6.75)	1.0 (5.1)	−0.76 to 2.76	0.15	0.3^*^	0.01	> 0.9^*^
Role limitations caused by physical health	86.0 (28.3)	92.7 (19.0)	6.6 (29.1)	−3.53 to 16.76	0.18	0.2^*^	93.6 (21.3)	94.3 (13.7)	0.7 (20.6)	−6.34 to 7.77	0.04	> 0.9^*^	0.12	0.2^*^
Role limitations caused by emotional problems	90.2 (22.5)	92.2 (21.8)	2.0 (18.2)	−4.40 to 8.33	0.17	0.7^*^	88.6 (22.8)	93.3 (17.7)	4.8 (21.6)	−2.66 to 12.18	0.27	0.2^*^	0.10	0.2^*^
Energy/Fatigue	69.1 (15.6)	72.5 (12.2)	3.4 (13.2)	−1.24 to 8.00	0.23	0.13	64.3 (16.5)	68.3 (13.9)	4.0 (12.7)	−0.35 to 8.35	0.17	0.11	0.03	0.9
Emotional well‐being	80.1 (13.0)	84.2 (11.2)	4.1 (9.7)	0.73 to 7.51	0.44	**0.013**	78.1 (11.3)	79.9 (11.0)	1.8 (8.6)	−1.12 to 4.78	0.23	0.2	0.13	0.3
Social functioning	89.3 (15.7)	91.9 (12.6)	2.6 (12.2)	−1.69 to 6.84	0.18	0.3^*^	92.9 (13.0)	93.6 (16.2)	0.7 (19.9)	−6.11 to 7.54	0.15	0.6^*^	0.00	> 0.9^*^
Pain	78.7 (17.4)	83.1 (17.7)	4.4 (16.2)	−1.26 to 10.08	0.28	0.089	85.1 (13.5)	86.9 (15.5)	1.9 (12.1)	−2.29 to 6.00	0.15	0.2	0.09	0.5
General health perception	72.7 (15.3)	74.9 (14.7)	2.2 (10.2)	−1.34 to 5.75	0.28	0.2	70.3 (14.2)	72.9 (12.8)	2.6 (10.2)	−0.92 to 6.07	0.26	0.2	0.01	> 0.9
Health change^a^	53.7 (15.2)	55.2 (14.8)	1.5 (10.6)	−2.21 to 5.15	0.14	0.4	54.3 (17.7)	56.4 (19.5)	2.1 (11.1)	−1.68 to 5.97	0.19	0.4	0.03	> 0.9

*Note*: Oat group *n* = 34; rice group *n* = 35. Higher score in RAND‐36 refers to better health. The bolded values present significances at the level *p* < 0.05, *p* values obtained from linear mixed model, *p*_time_ standing for within‐group change over time and *p*_group × time_ for between‐group difference in changes (interaction: group × time).

Abbreviations: 95% CI, confidence interval; ES, Wilcoxon effect size (*r*); SD: standard deviation.

^a^ The result from question no. 2 regarding the difference in health compared to that of 1 year ago.

* *p* value from the Wilcoxon signed rank test due to skewed data.

## 4 Discussion

Although the necessity of a GFD for CD patients is well established [63, 64, 65, 66], only a few previous studies have been conducted on the health effects of an LGD on volunteers without CD. Studying the health impact of LGDs on the general population is also important, as CD patients and gluten sensitive people can respond metabolically differently to dietary changes [42]. The diet, in addition to factors such as individual genetics, lifestyle, and physical activity, influences the risk factors for cardiometabolic diseases [30]. Evidence of low‐gluten intake increasing the risk of type 2 diabetes in non‐CD individuals has also been described in a recent review [36]. Oats are increasingly recognized for their versatile use as a naturally gluten‐free ingredient, which calls for comparative studies of oats with more traditional gluten‐free ingredients, such as rice. Previous studies have focused on effects mediated by either GFD or oats, and thus, to our knowledge, this is the first study to examine an LGD with oats or rice as the main cereal source and to compare their health effects on metabolically challenged individuals without CD. The results of the present study indicate that an LGD with > 4 portions of oat products and approx. seven grams of β‐glucan a day can achieve a reduction in the waist circumference, as well as LDL‐C and TC levels. In addition, the dietary intake stayed unchanged for most macro‐ and micronutrients, and the lowest level of the fiber recommendation (> 25 g/day) was reached. The gut symptoms were also found to be improved, and the perceived well‐being was maintained. Decreased body weight and waist circumference, as seen in this study, lower the risk for cardiometabolic diseases [67]. Similar findings in non‐CD patients have also been reported after a GFD [37, 38, 41, 42] or after an oat supplementation intervention, regardless of the background diet [40]. Increased whole‐grain consumption has been shown to reduce body weight [68], which can partly explain the positive changes within the oat group. Within the rice group, a significant reduction in anthropometrics was likely a result of a decreased energy intake, as observed in the food records. Although there was no difference between the two groups in self‐reported energy intake, waist circumference was reduced more with rice than with oats. Interestingly, significant weight loss on an LGD might be partly mediated by increased thermogenesis and dietary fiber quality [38], but the effect should be confirmed by further studies.

In addition to the anthropometric measures, the current study revealed that an oat‐rich LGD reduced LDL‐C level more than a rice‐rich LGD, which manifests the well‐known cholesterol reducing effects of oat β‐glucan [12, 13, 40]. There were also positive changes in TC within the oat group. Our findings regarding TC and LDL‐C are in accordance with a previous study, in which participants with mild hypercholesterolemia consumed 80 g of either oats (3 g β‐glucan in the oat group) or rice, daily for 45 days as part of their regular diet (without gluten restriction) [45]. The study found a significant decrease in the TC, LDL‐C, and non‐HDL‐C levels in the oat group compared to the rice group [45]. In our study, no significant reduction in TC within the rice group was detected, even though the implementation of an LGD for healthy adults was previously found to reduce TC and LDL‐C [38]. Reyna‐Villasmil et al. [43] found that 6 g of β‐glucan daily for 8 weeks increased HDL‐C concentration by 27.8% in hypercholesterolemic individuals. Such an effect was not seen in our 6‐week study, despite a relatively high β‐glucan consumption. In the cross‐sectional study by Kim et al. [37] an increased HDL‐C concentration was seen after 1 year on a GFD. Another 8‐week intervention study involving participants with moderate hypercholesterolemia and a low cardiovascular risk profile found a reduction of the TC and LDL‐C but not blood pressure with an intake of 3 g of β‐glucan [44]. In our study, we did not observe any significant changes in blood pressure, triacylglycerols, free fatty acids, glucose, or insulin, which is mostly in accordance with other studies investigating the impact of GFD or LGD on non‐CD patients [34, 36, 37, 41, 69]. Similar clinical results were also reported in a recent meta‐analysis on oat supplementation interventions [40]. The well‐known effects of oat β‐glucan on glucose metabolism might be more visible in postprandial glycemic and insulin peaks rather than in fasting values [45], which claims the need for further postprandial studies. The current view is that β‐glucan affects cholesterol homeostasis also by modulating the gut microbiota and not only by producing gels [70, 71]. An untargeted metabolomic study has also suggested different metabolic pathways, such as glycerophospholipid, sphingolipid, alanine, aspartate and glutamate, and retinol metabolism, to be behind the cholesterol‐lowering effects of oats [45]. Thus, not only the oat β‐glucan and its gel‐forming capabilities, but also its phytochemicals, soluble fibers, and their effects on microbiota potentially influence the cardiometabolic health, directly and indirectly [40]. Further studies on metabolomics and changes in microbiota, which affect bile acid and short‐chain fatty acid production, would provide broader insights into the cholesterol lowering mechanisms of oats as part of an LGD.

The compliance with the study diet was regarded as being successful as the participants fulfilled the target of 4 portions per day in both diet groups. As in previous clinical studies [41, 42, 69], we provided suitable products and guidance to follow the diet. In Finland, there are more versatile oat products available on the market compared to rice products, which was seen in the product usage of the participants. The phone discussion in the third week and the product diaries helped with the assessment of the adherence to the diet. Other studies have evaluated participants’ dietary adherence through questionnaires [42, 69], analysis of serum alkylrecinols [38], or discussions during weekly visits [41].

The dietary intake during the intervention differed significantly between the groups. The carbohydrate intake increased in the rice group without affecting the sugar intake. Oats are most often consumed as whole grain and are a good source of fiber [72]. Indeed, the largest difference between the diets was seen in the fiber intake that was considerably higher in the oat group compared to the rice group. Low fiber intake on an LGD has been reported in healthy Chinese students [73] and generally on GFD [41, 74–76]. The intake of zinc, magnesium, and iron decreased in the rice group and tended to increase in the oat group. Rice is mainly consumed as white rice that contains only part of the minerals of brown rice [22], while oats are a good source of minerals, especially iron, zinc, magnesium and phosphorus [6, 72]. Interestingly, folate intake decreased within both the groups below the recommended daily intake (330 µg/day) [77]. This finding is unusual because Finnish oats are known to be a relatively good source of folate [78]. The baseline folate intake reflected in their food record was found to be rather high (> 300 µg/day) compared to the average intake in the Finnish population (> 200 µg/day) [10], due to high consumption of folate‐rich foods such as liver and faba beans by certain participants. The reduction in the folate intake as found in their 6th week food record might also be due to the study products and LGD replacing their previous diet. This change in the diet might have also affected the intake of other micronutrients, for example, a reduction in vitamin C intake was observed in the oat group. Previously, a decrease in the magnesium intake during a 6‐month GFD intervention was reported in non‐CD patients [69]. Moreover, in a study comparing GF and gluten‐containing menus, except for the lower sodium content, the dietary quality of the GF menus was found to be inferior with a significantly lower content of protein, folate, magnesium, potassium, and vitamin E, and a trend towards lower calcium, and higher total fat content [76]. In the meta‐analysis part of the same study [76], CD patients on a GFD (not specified to contain oats) were found to have a higher total daily energy and total fat intake, and a lower fiber and folate intake than the controls. In our study, similar trends were seen in folate intake in both the groups, and in fiber, magnesium, potassium, and vitamin E intake only in the rice group. Furthermore, the absolute intake of protein (g/day) decreased significantly in the rice group; however, the proportional intake of protein (E%) was unchanged.

There were more reported bowel movements and a higher number of normal‐type stools with oats than with rice. Although not significant, the rice group reported in their defecation record a higher frequency of harder stool consistency and days without defecation compared to the oat group. Similarly, the rice group also had increased constipation and diminished diarrhea GSRS scores, which were found to be significant. Indeed, low fiber intake is linked to a higher occurrence of constipation and gastrointestinal symptoms, both of which are commonly present in untreated and treated CD patients [74, 79]. Improvement of the GSRS scores was found within both the groups, but not between the groups. Within the oat group, the scores for abdominal pain and reflux improved. This is in accordance with a previous study [80] where ulcerative colitis patients added 60 g of oat bran to their diet for 12 weeks and improved their abdominal and reflux GSRS scores. However, in another study [81], oat bran improved the indigestion score only after 16 weeks. The improvement in abdominal pain, more normal‐type stools, and increased bowel movements might result from oat fibers’ ability to increase the viscosity of the stool mass and reduce the colonic distension [82, 83, 84]. Decreased BMI and a higher whole‐grain consumption might have improved the reflux score, as they have been shown to manage gastroesophageal reflux disease [85]. The higher fiber consumption in the oat group can also regulate digestion and reduce acid reflux, and oatmeal can provide a soothing effect on the stomach lining [85]. The indigestion subcategory of the GSRS includes questions about bloating and flatulence that can be associated with the effects of fiber on the stool mass. Considering the low fiber intake in the rice group, the improved indigestion symptoms are reasonable. LGDs have been shown to be more efficient in improving bloating compared to high‐gluten diets [38], which can also explain the borderline significant improvement in indigestion within the oat group. Previously, gluten‐sensitive participants have experienced improved gastrointestinal symptoms with an LGD, but no differences in perceived health when compared to the control group [39]. Our findings are similar, since no significant changes in perceived health were observed between the groups according to the RAND‐36 questionnaire, even if the total score improved within both groups. Interestingly, there was an improvement of emotional well‐being within the oat group, which may be explained by previously discovered links between β‐glucan and the gut‐brain axis [15].

The strength of this study is the application of LGDs with either oats or rice in a real‐life setting with available commercial products and alongside the individual diet habits of the participants. Although the rice products were not identical to oats regarding the nutritional quality, the product categories were well matched. This strengthened the aim of comparing oats and rice as part of an LGD. The adherence to the diet was good and well monitored by providing the products and following their use with a separate diary and the third‐week control. It is important to also hear the participants’ subjective perceptions [86]; therefore, the inclusion of the questionnaires on general (RAND‐36) and gastrointestinal health (GSRS) enabled us to understand the health effects more comprehensively. All the questionnaires and records were verified individually with every participant upon return, and the instructions were made clear by the trained study nurse and assistants. The participants were highly committed and motivated, as indicated by the very low drop‐out rate.

Naturally, this study also has certain limitations. Even after the extensive efforts in the recruitment process, the sample size remained smaller than hoped for according to the power calculation. It was challenging to find suitable participants after the pandemic and without medication for high cholesterol. The power calculation was performed based on total cholesterol reduction, which indeed did not differ between the groups at the end of the intervention. However, LDL‐C reduced significantly, indicating the effect of the diet even with the number of participants that completed the study. Double blinding was not feasible due to the design of the study. We cannot totally discount other lifestyle factors affecting the results, since the participants may have paid more attention to their lifestyle after being recruited in the study, and had positive expectations of the outcomes, as also hypothesized in other studies [38, 45, 46, 87]. This can potentially explain the significant change in the RAND‐36 total score (assessing perceived well‐being) within the groups, but not between the groups. The implementation of the study diet into the participants’ own habitual diet and lifestyle was challenging in some cases, especially the participants in the rice group needed additional advice to ease constipation symptoms. However, even after following the advice of including more vegetables and fruits in the diet, their constipation symptoms did not improve, as seen in their GSRS score. It is notable that the food database used to evaluate diet quality did not include all of the particular study products, and the food record method could not reliably evaluate other micronutrients than those reported. Thus, the micronutrient results have to be interpreted with caution. Underreporting in the food records is more common with overweight people [88], and, despite personal verification of the records with each participant individually, relying solely on self‐reporting is a limitation. Not being able to measure the exact gluten intake of the participants, due to the fact that it was not included in the national database, is a limitation in this study. Here, the LGD was achieved by limiting the gluten sources. Analysis of the biomarkers of grains, such as alkylrecinols from blood, could have confirmed the compliance and helped to estimate gluten intake, as has been previously reported [38, 89].

According to our results, oats are a crucial part of an LGD in terms of nutritional quality. The overall dietary intake of the rice group was negatively affected during the 6‐week intervention, especially for fiber. Thus, a white rice‐based LGD could eventually lead to nutritional challenges and would not be considered a healthy choice. In conclusion, this study shows that oat‐based LGD improves both clinical markers for cardiometabolic health and perceived gastrointestinal symptoms in metabolically challenged volunteers. Our study suggests that oats are a particularly good cornerstone in LGDs and may prevent the risk of metabolic disease.

## Conflicts of Interest

The authors declare no conflicts of interest.

## Supporting information



Supporting information

## Data Availability

The data that support the findings of this study are available from the corresponding author upon reasonable request. The data are not publicly available due to privacy or ethical restrictions.
