# Supplementary material for: Comparison of Low‐Gluten Diets Rich in Oats or Rice—A 6‐Week Randomized Clinical Trial With Metabolically Challenged Volunteers
Source: Mol Nutr Food Res. 2025 May 7;69(12):e70076. doi: 10.1002/mnfr.70076 (PMC12189175; doi:10.1002/mnfr.70076)
Supplement: Supplementary file 1 — Supporting information [file MNFR-69-e70076-s001.pdf]

Supporting Information to the

Comparison of low-gluten diets rich in oats or rice – a 6-week randomized clinical trial with metabolically challenged volunteers

Enni Mannila[1] (ORCID: 0000-0002-8199-8137), Petrus Nuotio[2] (ORCID: 0000-0002-6915-5153), Anni Kuosmanen[2], Suchetana De Storvik[2], Anna Kårlund[1] (ORCID: 0000-0003-4078-1035), Aija Jukkara[2], Milla-Maria Tauriainen[3], Johanna Närväinen[4], Marjukka Kolehmainen[2#](ORCID: 0000-0002-3770-2538), Kaisa M. Linderborg[1#] (ORCID: 0000-0003-1977-7322)

[#]equal contribution, Professor Kaisa Linderborg, Itäinen Pitkäkatu 4 C, 20520 Turku, Finland,

kaisa.linderborg@utu.fi

Professor Marjukka Kolehmainen, Yliopistonranta 1C, 70210 Kuopio, Finland,

marjukka.kolehmainen@uef.fi

[1]Food Sciences, Department of Life Technologies, University of Turku, Turku, Finland
[2]Institute of Public Health and Clinical Nutrition, School of Medicine, Faculty of Health Sciences, University of Eastern Finland, Kuopio, Finland
[3]Department of Medicine, Endoscopy Unit, Kuopio University Hospital, Kuopio, Finland
[4]VTT Technical Research Centre of Finland, Kuopio, Finland

Supporting Information Table 1. The provided study products. If participant had consumed other oat or rice products than the study products, they were categorized as "Other".

Study products	1 portion	Energy (kcal)
**Oat**		
flakes	1 dl (40 g) / 230 g as oatmeal	146
Cookable meal grains	0,75 dl (60 g)	216
Seasoned meal grains	1/4 bag (63 g)	90
Granola	1 dl (50 g)	210
Snack bar	1 pcs (30 g)	138
**Rice**		
flakes	¾ dl (40 g), / 230 g as rice porridge	143
Rice	1 dl (cooked)	114
Seasoned rice bag cooked with unflavored regular rice	1 dl (cooked)	110
Rice crispies	2 dl (30 g)	118
Rice cake	4 pcs (30 g in total)	127
**Other suitable products (self-bought)**		
**Oat**		
bread (100% oat)	1 slice (32 g)	78[a]
bran	3 tbsp. (27 g)	102[a]
pasta (100% oat)	0,5 dl (33 g)	123
**Rice**		
homemade rice bread (according to a given recipe)	1/6 of the recipe	
noodles (from rice flour)	1 ball (45 g)	159[a]
gluten free pasta (from rice flour)	1 dl (cooked)	

a) From Fineli food database https://fineli.fi/fineli/en/

Oat group: Study products were 58.1%, suitable products 34.8% (oat bread) and 3.3% (bran, pasta), and the other 3.8% of the reported daily portions.

Rice group: Study products were 94.1%, suitable products were 2.3% and the other were 3.6% of the reported daily portions.

Rice bread recipe (1 baking sheet; 1/6 baking sheet = 185 g = 1 daily portion of rice)

4 dl water, 2 dl porridge rice, 50 g margarine, 200 g low-fat crème fraîche (Finnish: *kermaviili*), 6 dl milk/soya drink/other liquid, 2 eggs, 2 dl buckwheat flakes, 1.5 dl rice flakes, 1 tsp. salt. Porridge rice and margarine cooked in water. Mixed all ingredients and spread on the baking sheet. Baked at 225–250 °C approx. 20 minutes.

Supporting Information Table 2. Other reported products consumed in addition to the study products and suitable products. These reflect 3.8% of all products used within the oat group and 3.6% within the rice group.

OAT	%	RICE	%
spoonable oat product	54.4	rice drink	37.3
home-made oat pastries	16.1	rice pudding	32.4
oat drink/cream	11.8	rice (crisp)bread	12.2
oat muesli and cereals	6.5	chocolate with rice	11.4
oat protein product	4.0	gluten-free pizza made with rice flour	2.9
oat ice cream	3.4	rice ice cream	1.3
other oat snack bar	2.4	gluten-free vegetarian pastry	1.3
oat flour	0.7	rice flour	0.4
oat crispbread	0.3	stuffed cabbage leaves with rice	0.4
semolina pudding from oats	0.3	black rice	0.4
total	100.0		100.0

Supporting Information Table 3. The nutritional intake of the oat and rice group in the baseline and after the 6-week intervention calculated from the 4-day food records. The values are presented as mean (standard deviation) with 95% confidence interval for the change.

	Oat						Rice							
	Baseline (n=34)	Week 6 (n=34)					Baseline (n=35)	Week 6 (n=34)						
	mean (SD)	mean (SD)	Δ (SD)	Δ 95% CI	ES_{time}	p_{time}	mean (SD)	mean (SD)	Δ (SD)	Δ 95% CI	ES_{time}	p_{time}	ES group × time	$p_{group \times time}$
Total energy, kcal/day	2090 (442)	2089 (401)	-1 (462)	-162 to 160	0.00	>0.9	2272 (569)	2117 (560)	-186 (380)	-318 to -53	0.43	**0.011**	0.19	0.10
Total energy, kJ/day	8750 (1851)	8744 (1679)	-6 (1932)	-680 to 668	0.00	>0.9	9510 (2384)	8863 (2345)	-778 (1589)	-1332 to -223	0.43	**0.011**	0.19	0.10
Protein, g/day	89.17 (28.82)	88.24 (21.94)	-0.94 (24.96)	-9.65 to 7.77	0.04	>0.9	90.48 (24.55)	83.56 (28.71)	-7.83 (19.55)	-14.65 to -1.00	0.37	**0.017**	0.17	0.090
Protein, E%	17.20 (3.24)	17.17 (2.68)	-0.03 (2.49)	-0.90 to 0.84	0.03	>0.9	16.36 (2.81)	15.98 (3.26)	-0.27 (2.19)	-1.03 to 0.50	0.07	0.4	0.04	0.6
Carbohydrate, g/day	210.85 (53.93)	202.59 (42.77)	-8.25 (57.74)	-28.40 to 11.89	0.18	0.5	238.05 (68.19)	246.34 (67.47)	4.50 (45.37)	-11.33 to 20.33	0.16	0.5	0.17	0.3
Carbohydrate, E%	41.12 (7.07)	39.65 (6.01)	-1.47 (6.52)	-3.75 to 0.81	0.20	0.2	42.53 (5.12)	47.49 (5.88)	4.79 (6.14)	2.65 to 6.93	0.64	**< 0.001**	0.44	**<0.001**
Total sugar, g/day	92.5 (29.3)	92.6 (31.5)	0.121 (33.4)	-11.5 to 11.8	0.09	>0.9	100 (40.4)	94.3 (38.6)	-8.13 (19.7)	-15.0 to -1.27	0.44	**0.027**	0.21	0.3
Total sugar, E%	18.0 (4.60)	18.0 (5.09)	-0.0299 (5.13)	-1.82 to 1.76	0.03	>0.9	17.8 (5.86)	17.8 (5.03)	-0.305 (3.25)	-1.44 to 0.827	0.10	0.7	0.05	0.9
Fiber, g/day	23.39 (7.14)	26.58 (8.50)	3.19 (8.42)	0.26 to 6.13	0.35	**0.048**	25.35 (8.44)	17.40 (6.09)	-8.35 (7.11)	-10.83 to -5.87	0.80	**<0.001**	0.61	**< 0.001**
Fiber, g/MJ	2.68 (0.64)	3.05 (0.78)	0.37 (0.69)	0.13 to 0.61	0.46	**0.006**	2.71 (0.79)	2.04 (0.75)	-0.68 (0.91)	-1.00 to -0.36	0.66	**<0.001**	0.59	**< 0.001**
Total fat, g/day	89.81 (24.52)	92.26 (25.20)	2.46 (25.15)	-6.32 to 11.23	0.03	0.6	97.86 (29.10)	79.26 (24.07)	-19.81 (24.85)	-28.48 to -11.14	0.66	**<0.001**	0.36	**< 0.001**
Total fat, E%	37.94 (6.82)	38.83 (5.80)	0.89 (7.26)	-1.65 to 3.42	0.12	0.4	37.96 (5.58)	32.95 (4.30)	-4.91 (5.92)	-6.97 to -2.84	0.68	**<0.001**	0.39	**< 0.001**

Saturated fatty acids, E%	13.79 (3.89)	13.69 (3.28)	-0.10 (3.57)	-1.35 to 1.14	0.02	>0.9	14.09 (3.36)	11.81 (3.10)	-2.17 (3.09)	-3.25 to -1.09	0.58	**<0.001**	0.30	**0.009**
Monounsaturated fatty acids, E%	13.79 (2.57)	14.54 (2.95)	0.76 (3.20)	-0.36 to 1.87	0.20	0.2	13.87 (2.55)	11.95 (1.85)	-1.93 (3.15)	-3.02 to -0.83	0.51	**<0.001**	0.37	**0.001**
Polyunsaturated fatty acids, E%	6.63 (1.76)	6.95 (1.99)	0.32 (1.97)	-0.37 to 1.01	0.11	0.4	6.27 (1.66)	5.98 (1.92)	-0.32 (2.00)	-1.02 to 0.37	0.21	0.3	0.16	0.2
Folate, µg/day	313.22 (72.25)	260.77 (80.31)	-52.46 (84.63)	-81.98 to -22.93	0.54	**< 0.001**	300.01 (102.66)	260.31 (92.83)	-42.81 (113.74)	-82.49 to -3.12	0.42	**0.017**	0.02	0.6
Salt (NaCl), mg/day	7.79 (2.44)	7.79 (2.25)	0.01 (2.62)	-0.91 to 0.92	0.03	>0.9	8.55 (2.23)	8.99 (2.48)	0.32 (2.01)	-0.38 to 1.02	0.10	0.3	0.03	0.5
Magnesium, mg/day	382.33 (118.35)	417.14 (101.58)	34.81 (113.65)	-4.85 to 74.46	0.22	0.075	398.74 (94.41)	375.23 (101.02)	-28.29 (67.51)	-51.84 to -4.73	0.42	**0.020**	0.29	**0.007**
Calcium, mg/day	1034.38 (431.05)	1006.94 (438.97)	-27.44 (354.18)	-151.01 to 96.14	0.12	0.6	1104.63 (363.05)	1031.74 (355.56)	-91.69 (274.70)	-187.54 to 4.16	0.22	0.069	0.05	0.5
Iron, mg/day	12.44 (3.56)	13.54 (2.72)	1.10 (3.58)	-0.15 to 2.35	0.29	0.056	13.07 (3.79)	11.93 (3.57)	-1.33 (3.07)	-2.40 to -0.26	0.39	**0.020**	0.34	**0.003**
Potassium, mg/day	3810.58 (1048.92)	3559.01 (950.00)	-251.57 (919.55)	-572.42 to 69.28	0.30	0.2	3798.75 (1053.20)	3466.93 (1078.15)	-387.67 (717.41)	-637.98 to -137.35	0.48	**0.003**	0.05	0.5
Zinc, mg/day	12.35 (4.41)	13.28 (3.36)	0.94 (3.85)	-0.41 to 2.28	0.29	0.074	12.77 (3.67)	11.42 (3.89)	-1.53 (2.73)	-2.48 to -0.58	0.48	**0.004**	0.38	**0.001**
Vitamin E, mg/day	13.20 (4.39)	11.82 (4.30)	-1.38 (4.56)	-2.97 to 0.21	0.29	0.070	12.85 (4.67)	11.41 (3.84)	-1.65 (4.36)	-3.17 to -0.13	0.48	**0.033**	0.06	>0.9
Vitamin C, mg/day	144.83 (59.36)	107.24 (55.98)	-37.59 (66.06)	-60.64 to -14.54	0.50	**0.002**	131.19 (71.92)	121.78 (76.90)	-12.01 (61.54)	-33.48 to 9.46	0.18	0.2	0.18	0.11

Baseline n = 69, week 6 n = 68. SD: standard deviation, 95% CI: confidence interval, E%: nutrient intake as a percentage of total energy intake, ES: Wilcoxon effect size (*r*), p-values obtained from linear mixed model, p_{time} standing for within-group change over time and $p_{group \times time}$ for between-group difference in changes (interaction: group × time).

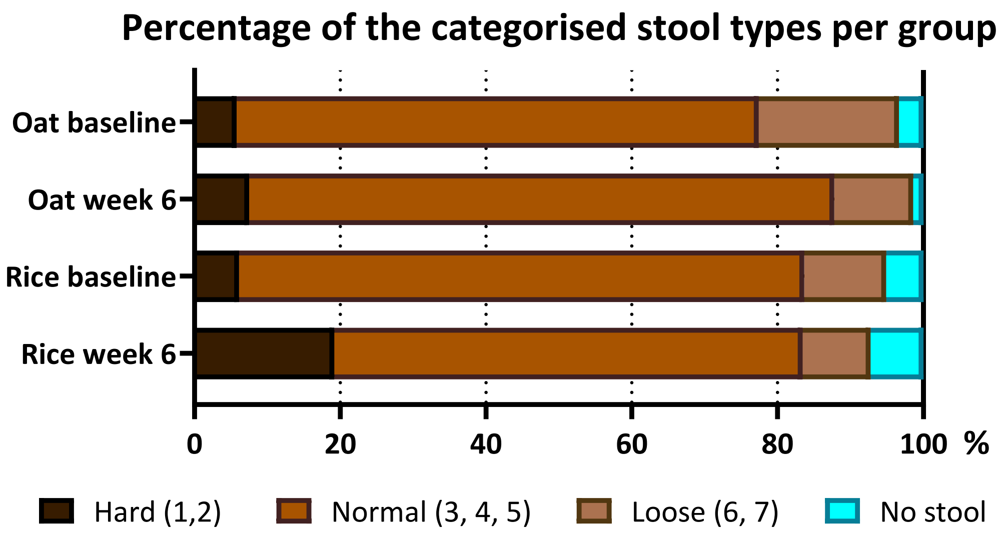

Supporting Information Figure 1. The changes of stool types within and between the groups presented as percentages. The numbers in brackets represent the stool types of the Bristol scale that were grouped in each category. If the participant reported no defecation on any of the four days, that case was categorized as “no stool”. The reporting of normal stool during the intervention was significantly different between the groups (interaction $p = 0.010$), within the oat group, normal stool tended to increase ($p = 0.082$) and within the rice group, it tended to decrease ($p = 0.056$). However, these changes were considered small according to the effect sizes ($r < 0.30$).

Supporting Information Table 4. Results of Gastrointestinal Symptoms Rating Scale (GSRS) questionnaire scores presented as mean (standard deviation) with 95% confidence interval for the change.

	**Oat**						**Rice**							
	**Baseline**	**Week 6**					**Baseline**	**Week 6**						
GSRS Score	**mean (SD)**	**mean (SD)**	**Δ (SD)**	**Δ 95% CI**	**ES_{time}**	**p_{time}**	**mean (SD)**	**mean (SD)**	**Δ (SD)**	**Δ 95% CI**	**ES_{time}**	**p_{time}**	**$ES_{group \times time}$**	**$p_{group\ x\ time}$**
Total	11.3 (5.9)	9.0 (4.5)	-2.3 (5.9)	-4.4 to -0.3	0.42	**0.033**	11.0 (4.8)	10.1 (4.4)	-0.9 (4.9)	-2.5 to 0.8	0.18	0.3	0.12	0.3
Abdominal pain	1.6 (1.3)	1.1 (1.3)	-0.5 (1.7)	-1.1 to 0.1	0.32	**0.018**	1.4 (1.1)	1.4 (1.3)	-0.03 (1.2)	-0.4 to 0.4	0.06	0.6	0.15	0.12
Indigestion	4.6 (1.9)	3.7 (1.7)	-0.8 (2.4)	-1.7 to 0.0	0.33	0.050	4.9 (2.1)	3.7 (1.9)	-1.3 (2.7)	-2.2 to -0.4	0.47	**0.008**	0.11	0.4
Reflux	1.4 (1.4)	0.9 (1.2)	-0.5 (1.0)	-0.9 to -0.2	0.48	**0.005**	0.7 (1.1)	0.5 (0.9)	-0.2 (0.9)	-0.5 to 0.1	0.13	0.4	0.19	0.3
Constipation	1.8 (1.7)	1.6 (1.6)	-0.2 (1.1)	-0.6 to 0.2	0.21	0.2	2.0 (1.7)	3.2 (2.0)	1.2 (2.0)	0.5 to 1.9	0.53	**0.001**	0.40	**<0.001**
Diarrhea	2.0 (2.0)	1.8 (1.3)	-0.2 (2.1)	-1.0 to 0.5	0.06	0.7	2.0 (1.7)	1.4 (1.3)	-0.6 (1.4)	-1.1 to -0.1	0.40	**0.042**	0.15	0.3

Oat group n = 34; Rice group n = 35. Higher score in GSRS refers to more symptoms. SD: standard deviation, 95% CI: confidence interval, ES: Wilcoxon effect size (*r*), p-values obtained from linear mixed model, p_{time} standing for within-group change over time and $p_{group\ x\ time}$ for between-group difference in changes (interaction: group × time).
